# Mitochondrial Respiration Regulates Adipogenic Differentiation of Human Mesenchymal Stem Cells

**DOI:** 10.1371/journal.pone.0077077

**Published:** 2013-10-18

**Authors:** Yanmin Zhang, Glenn Marsboom, Peter T. Toth, Jalees Rehman

**Affiliations:** 1 Section of Cardiology, Department of Medicine, University of Illinois at Chicago, Chicago, Illinois, United States of America; 2 Department of Pharmacology, University of Illinois at Chicago, Chicago, Illinois, United States of America; 3 Imaging Center, Research Resources Center, University of Illinois at Chicago, Chicago, Illinois, United States of America; Instituto Butantan, Brazil

## Abstract

Human mesenchymal stem cells (MSCs) are adult multipotent stem cells which can be isolated from bone marrow, adipose tissue as well as other tissues and have the capacity to differentiate into a variety of mesenchymal cell types such as adipocytes, osteoblasts and chondrocytes. Differentiation of stem cells into mature cell types is guided by growth factors and hormones, but recent studies suggest that metabolic shifts occur during differentiation and can modulate the differentiation process. We therefore investigated mitochondrial biogenesis, mitochondrial respiration and the mitochondrial membrane potential during adipogenic differentiation of human MSCs. In addition, we inhibited mitochondrial function to assess its effects on adipogenic differentiation. Our data show that mitochondrial biogenesis and oxygen consumption increase markedly during adipogenic differentiation, and that reducing mitochondrial respiration by hypoxia or by inhibition of the mitochondrial electron transport chain significantly suppresses adipogenic differentiation. Furthermore, we used a novel approach to suppress mitochondrial activity using a specific siRNA-based knockdown of the mitochondrial transcription factor A (TFAM), which also resulted in an inhibition of adipogenic differentiation. Taken together, our data demonstrates that increased mitochondrial activity is a prerequisite for MSC differentiation into adipocytes. These findings suggest that metabolic modulation of adult stem cells can maintain stem cell pluripotency or direct adult stem cell differentiation.

## Introduction

Human mesenchymal stem cells (hMSCs) are adult multipotent stem cells that can be isolated from several tissues including bone marrow and adipose tissue. Initially, they were identified as fibroblast precursors that are present in the bone marrow [1], [2] and subsequently their mesodermal differentiation potential into osteoblasts, chondrocytes and adipocytes was demonstrated [3]. Cell-based regenerative therapies using hMSCs are emerging as promising therapeutic approaches in a variety of vascular illnesses such as myocardial infarction [4], [5], [6] and ischemic cardiomyopathy [7]. Knowledge of the specific mechanisms that regulate the survival and the differentiation of hMSCs is critical to develop feasible clinical therapies.

Mitochondria generate ATP through oxidative phosphorylation and also release multiple signaling molecules, including reactive oxygen species (ROS) and calcium [8], [9], [10]. Recent studies have demonstrated the importance of mitochondrial metabolism in regulating stem cell biology. For example, embryonic stem cells increase their mitochondrial activity during differentiation [11] and the resulting increase in oxidative phosphorylation can impact the differentiation, as cells treated with a mitochondrial uncoupler during differentiation continued to form teratomas [12]. In MSCs, a marked increase in mitochondrial mass and oxygen consumption was observed upon osteogenic differentiation, which is associated with the upregulation of the mitochondrial biogenesis regulator Peroxisome proliferator-activated receptor gamma coactivator 1-alpha (PGC-1α) and anti-oxidant enzymes such as catalase and superoxide dismutase 2 (SOD2) [13]. Similarly, an increase in the abundance of mitochondrial oxidative phosphorylation supercomplexes during adipogenic differentiation of MSCs has been described [14]. Moreover, mitochondrial transfer from MSCs can increase alveolar epithelial cell survival after lipopolysaccharide challenge [3] and fusion between MSCs and cardiomyocytes leads to the development of cells with a cardiac progenitor phenotype, which is dependent on the transfer of functional MSC mitochondria [15]. These studies indicate that mitochondria play an important regulatory role in determining the therapeutic efficacy and differentiation capacity of MSCs.

In our study, we investigated mitochondrial biogenesis and activity during adipogenic differentiation of hMSCs, and we also studied whether mitochondrial function could impact the adipogenic differentiation of hMSCs.

## Materials and Methods

### Cell Culture

Human mesenchymal stem cells (hMSCs) were obtained from the Tulane Center for Gene Therapy (Tulane University, New Orleans, LA) and cultured in hMSC growth medium (16.5% FBS in α-MEM supplemented with 2 mM L-glutamine) or adipogenic differentiation medium as described previously [16]. Basically, 500 µM Isobutylmethylxanthine (IBMX), 1 µM Dexamethasone, 50 µM Indomethacin and 5 µg/ml Insulin (all from Sigma Aldrich, St Louis, MO) were added to hMSC growth medium to induce adipogenic differentiation.

### Oil Red O Staining

hMSCs were cultured in adipogenic differentiation medium for 7 days or 21 days. To confirm adipogenic differentiation, cells were fixed in buffered formalin for 1 hour at room temperature, followed by 20 minutes in 0.3% Oil Red O staining solution (Sigma Aldrich, St Louis, MO).

### Mitochondrial Staining

Mitochondria were stained with 100 nM MitoTracker Green FM (Invitrogen, Carlsbad, CA) for 20 minutes at 37°C in the dark. Cells were then washed and imaged immediately using a Zeiss LSM 510 META confocal microscope. For visualization of mitochondrial membrane polarization, cells were stained with 1 µg/ml JC-1 (5,5′,6,6′-tetrachloro-1,1′,3,3′-tetraethylbenzimidazolyl-carbocynanine iodide; Invitrogen, Carlsbad, CA) for 20 minutes at 37°C in the dark. Cells were then imaged using a Zeiss LSM 510 META confocal microscope.

### O_2_ Consumption Measurements

For oxygen consumption measurements in adherent cells, both the differentiated and undifferentiated hMSCs were seeded at 100,000 cells per well overnight. The oxygen consumption rate (OCR) and the extracellular acidification rate (ECAR) of adherent cells were measured at 37°C using a Seahorse XF24 Extracellular Flux Analyzer (Seahorse Bioscience, Billerica, MA, USA) as described previously [17], [18]. The uncoupler FCCP (Carbonyl cyanide 4-trifluoromethoxy phenylhydrazone; Sigma Aldrich, St Louis, MO) was used to measure maximal oxygen consumption rates.

### RNA Extraction and Real-time RT-PCR

Total RNA was extracted with the PureLink RNA Micro Kit (Invitrogen, Carlsbad, CA) according to the manufacturer’s instructions. The mRNA level of each gene was analyzed with real-time RT-PCR. Briefly, cDNA was synthesized from total RNA using the High-Capacity cDNA Reverse Transcription Kit (Applied Biosystems, Carlsbad, CA) and real-time RT-PCR was carried out on an Applied Biosystems 7900HT instrument. Primer sequences are available in Table S1.

### Flow Cytometry

Cells were stained with 500 nM MitoTracker Green FM (Invitrogen, Carlsbad, CA) for 30 minutes at 37°C in the dark, then washed and detached by trypsin and resuspended in staining buffer (BD Biosciences, San Jose, CA). The intensity of fluorescence was measured and analyzed on a FACSCalibur (BD Biosciences, San Jose, CA).

### ATP Assay

Cells were detached, counted and Adenosine 5′-triphosphate (ATP) levels were determined with a bioluminescent somatic cell assay kit (Sigma Aldrich, St Louis, MO) according to the manufacturer’s instructions.

### Mitochondrial Redox-sensitive GFP

A redox-sensitive GFP construct (roGFP2) targeted to the mitochondria (a kind gift of Dr. Remington, University of Oregon) [19] was transfected into hMSCs using Fugene HD transfection reagent (Roche, Indianapolis, IN). By using different excitation wavelengths (400 and 490 nm) and measuring emission at 535 nm, the redox status of cells was assessed (the higher the 400/490 nm ratio, the more oxidized the hMSCs). Images were acquired on a Zeiss AxioObserver Z1 microscope. Cells were perfused at room temperature with Krebs-Henseleit buffer solution at 21% O_2_ and 5% CO_2_.

### Immunofluorescence Staining

Cells were washed three times with PBS, fixed with 4% paraformaldehyde for 10 minutes at room temperature, and permeabilized with 0.2% Tween 20 in PBS for 10 minutes at room temperature. Cells were then washed in PBS, blocked in blocking buffer (DAKO, Carpinteria, CA) for 1 hour at room temperature, and subsequently incubated in a humidified chamber overnight at 4°C with monoclonal antibodies against TFAM (1∶50 dilution; Santa Cruz Biotechnology, Santa Cruz, CA), PDH subunit E1 alpha (1∶200 dilution; MitoSciences, Eugene, OR) or HIF-1alpha (1∶300 dilution; Novus Biologicals LLC, Littleton, CO). After 3 washes with buffer containing 10 mM Tris (pH 7.5), 100 mM NaCl and 0.1% Tween-20 (TBST), cells were incubated with secondary antibody in antibody diluent for 2 hours at room temperature. After washing with TBST, coverslips were mounted on microscopy slides using SlowFade Gold anti-fade reagent with DAPI (Invitrogen, Carlsbad, CA) and imaged with a Zeiss LSM 510 META confocal microscope.

### Immunoblotting

For immunoblot analysis, 20 µg whole-cell extracts from undifferentiated or 7-day adipogenic differentiated hMSCs were loaded onto a 4–15% Tris-HCl precast gel (BioRad, Hercules, CA). After electrophoresis, proteins were electro-blotted onto a nitrocellulose membrane (BioRad, Hercules, CA) and blocked with blocking buffer (Li-Cor, Lincoln, NE) for 1 hour at room temperature. The membranes were then probed with antibodies against TOM20 (1∶500, Santa Cruz Biotechnology, Santa Cruz, CA), SOD1 (1∶1000, Cell Signaling Technology, Danvers, MA), SOD2 (1∶1000, Santa Cruz Biotechnology, Santa Cruz, CA), Catalase (1∶1000, Cell Signaling Technology, Danvers, MA) and ß-actin (1∶10000, Abcam, Cambridge, MA) in blocking buffer overnight at 4°C. Fluorescently labeled secondary antibodies (1∶400, Invitrogen, Carlsbad, CA) were incubated for 2 hours at room temperature. After three washes with TBST for 10 minutes each, antibody binding was visualized with a BioRad Molecular imager FX Pro Plus (BioRad, Hercules, CA). Quantity One software was used for quantification of intensities.

### Small Interfering RNA (siRNA)

hMSCs were exposed to siTFAM (IDT, Coralville, Iowa) and scramble control. Three different siRNAs were tested and the siRNA with the most efficient siRNA was subsequently used during experiments.

### Statistical Analysis

Statistical analysis was performed using Graphpad Prism software. Inter-group differences between 2 groups were assessed by an unpaired Student’s t-test while an ANOVA with post hoc analysis using Tukey’s multiple comparison test was used for comparison amongst multiple groups. Data are presented as mean±SEM and p<0.05 was considered to be statistically significant. In the figures, *indicates p<0.05, **indicates p<0.01 and ***indicates p<0.001.

## Results

### Adipogenic Differentiation Enhances Mitochondrial Oxidation in hMSCs

**Figure 1:**
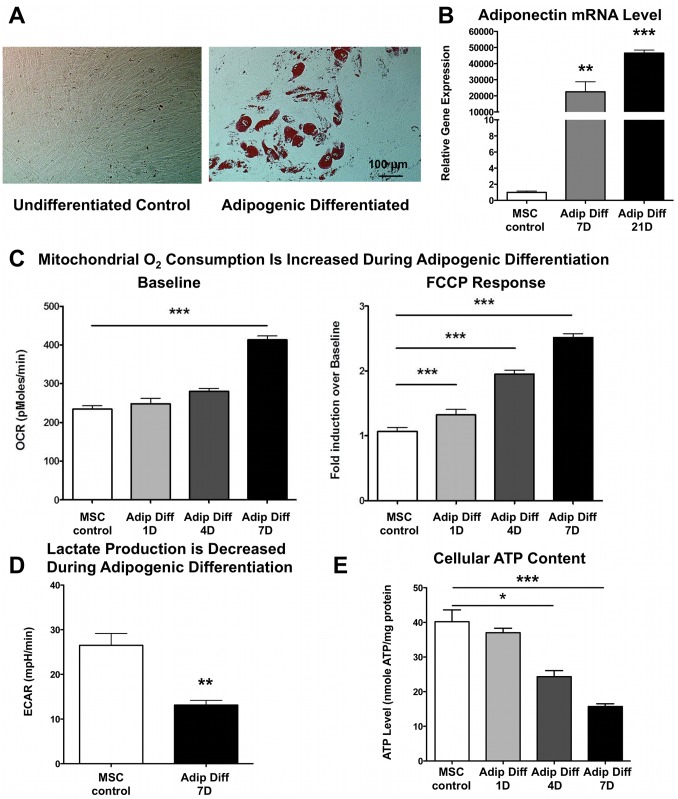
Adipogenic differentiation enhances mitochondrial oxidation in hMSCs. **A)** Bone marrow-derived hMSCs underwent adipogenic differentiation using 500 nM Isobutylmethylxanthine, 1 µM Dexamethasone, 50 µM Indomethacin and 5 µg/ml Insulin. Oil Red O staining was used to confirm the adipogenic differentiation of hMSCs at day 21 (n = 4 and representative pictures are shown). **B)** Real-time RT-PCR confirmed the upregulation of the adipogenic differentiation marker adiponectin (n = 5 for each group). **C)** Oxygen consumption rate (OCR) increases gradually during adipogenic differentiation. Furthermore, the maximal OCR as elicited by treatment with the mitochondrial uncoupler FCCP (2 µM) is also increased during adipogenic differentiation (n≥5 for each group). **D)** Lactate production was decreased after adipogenic differentiation, indicating decreased glycolysis upon differentiation (n = 9 for each group). **E)** Cellular ATP content normalized to total cellular protein decreased gradually during 7 days of adipogenic differentiation (n = 5 for each group).

Human mesenchymal stem cells were cultured in adipogenic differentiation medium and showed robust Oil Red O staining after 21 days, indicated by the presence of lipid droplets and therefore the formation of adipocytes (Figure 1A). The increased mRNA expression levels after 7 and 21 days of adiponectin, a protein hormone made by adipose tissue, confirmed these results (Figure 1B). Mitochondrial oxygen consumption almost doubled after 7 days of differentiation compared to undifferentiated hMSCs (Figure 1C). Moreover, we assessed maximal oxygen consumption with the mitochondrial uncoupler FCCP. Upon adipogenic differentiation, hMSCs exhibited a higher FCCP response, suggesting that they not only acquire higher baseline oxygen consumption but that they also exhibit a higher maximal oxygen consumption reserve capacity (Figure 1C). Concomitantly, lactate production decreased in the differentiated cells (Figure 1D), suggesting that there is a switch from glycolysis in undifferentiated hMSCs to oxidative phosphorylation in hMSCs undergoing adipogenic differentiation. Interestingly, the cellular ATP content decreased over time upon adipogenic differentiation despite the higher levels of oxygen consumption (Figure 1E).

### Mitochondrial Biogenesis Increases with Adipogenic Differentiation of hMSCs

**Figure 2:**
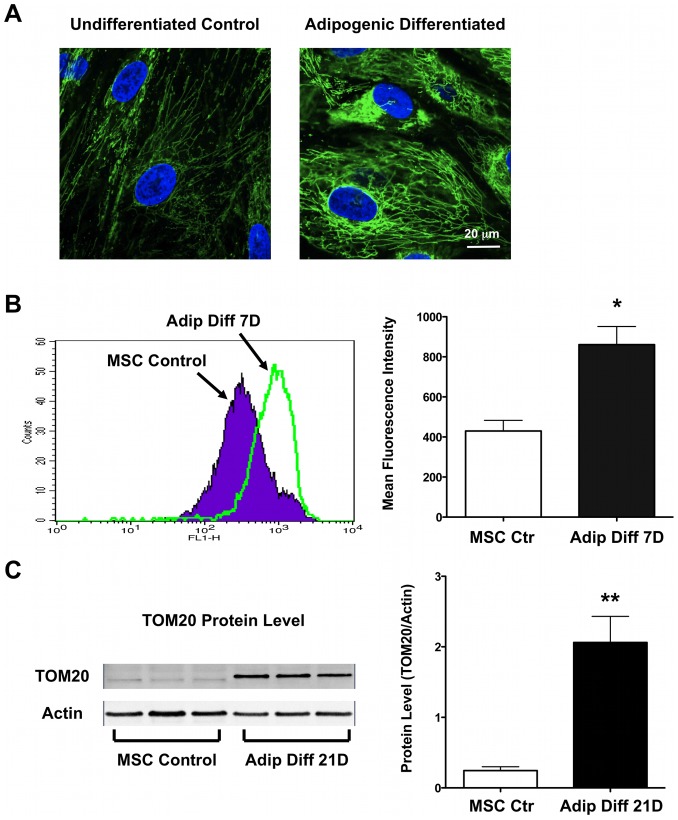
Mitochondrial biogenesis increased with adipogenic differentiation of hMSCs. **A)** Adipogenic differentiation was associated with a marked increase in mitochondrial mass, as demonstrated by increased MitoTracker Green staining. **B)** Flow cytometry measurement of MitoTracker Green staining confirmed the increase of mitochondrial mass as the mean fluorescence intensity is doubled after 7 days of adipogenic differentiation (n = 3 for each group). **C)** The protein levels of the mitochondrial outer membrane protein TOM20, a reliable marker of mitochondrial mass, showed a marked increase after adipogenic differentiation.

Since a significant increase of oxygen consumption was seen upon differentiation, we next measured mitochondrial biogenesis during adipogenic differentiation. MitoTracker Green stains mitochondria regardless of mitochondrial membrane potential and thus is a good marker to estimate mitochondrial mass. We observed increased MitoTracker Green staining after 7 days of adipogenic differentiation (Figure 2A) and confirmed this by flow cytometry (Figure 2B). In addition, immunoblotting for the outer mitochondrial membrane protein TOM20 confirmed a strong increase after 21 days of differentiation (Figure 2C). Pyruvate dehydrogenase (PDH) regulates the conversion of pyruvate into acetyl-CoA and thus is important for supplying carbon atoms for the Krebs cycle and for regulation mitochondrial activity. PDH expression was increased in differentiated hMSCs (Figure S1).

### Mitochondrial Membrane Potential and Reactive Oxygen Species Change with hMSC Adipogenic Differentiation

**Figure 3:**
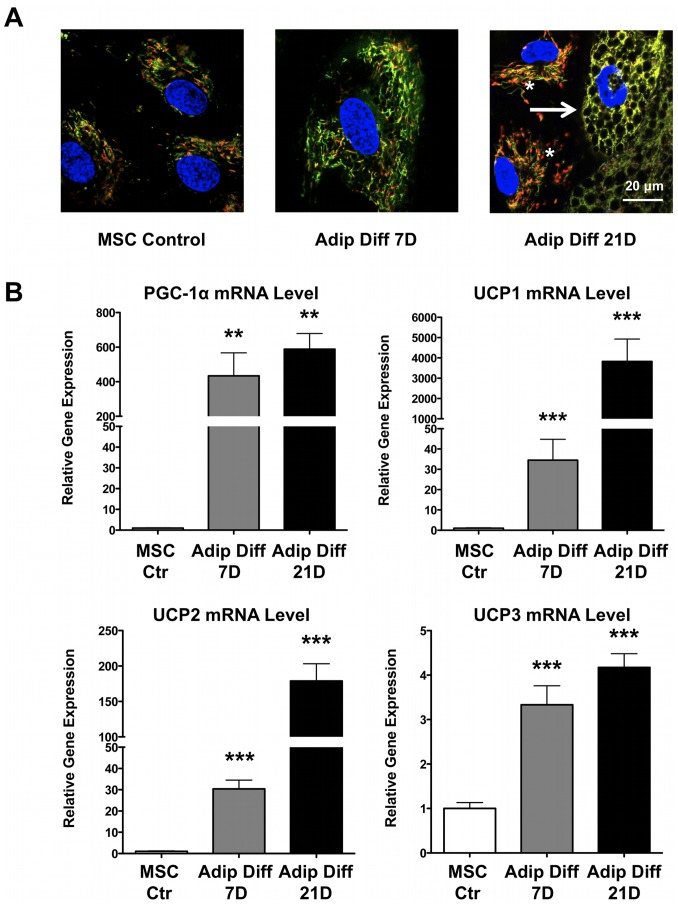
Mitochondrial membrane potential changed with hMSC adipogenic differentiation. **A)** JC-1 staining was used for the measurement of mitochondrial membrane potential. The ratio of red/green (and thus polarization) decreased with adipogenic differentiation. Note that not all cells were fully differentiated even after 21 days. Asterisks highlight the smaller, undifferentiated cells, while the arrow points at a larger and well-differentiated cell that contains multiple lipid droplets. Upon differentiation, mitochondrial depolarization (green color) was clearly present. **B)** Real-time RT-PCR data showed increased expression of PGC-1α and of the 3 uncoupling proteins (UCP1, 2, 3) following adipogenic differentiation (n = 5 for each group).

The potentiometric dye JC1 accumulates in the mitochondria. At low mitochondrial membrane potential (depolarized mitochondria), it is characterized by green fluorescence, while in the setting of higher mitochondrial membrane potential, JC1 forms aggregates that exhibit red fluorescence. Consequently, mitochondrial depolarization is indicated by a decrease in the red/green fluorescence intensity ratio. We compared JC1 staining in control hMSCs and cells after 7 and 21 days of adipogenic differentiation, and observed depolarization of mitochondrial membrane potential during differentiation (Figure 3A). Expression of the mitochondrial biogenesis regulator Peroxisome proliferator-activated receptor gamma coactivator 1 alpha (PGC-1α) and expression of the uncoupling proteins UCP1, UCP2 and UCP3 all increased during differentiation (Figure 3B). Since uncoupling proteins uncouple mitochondrial oxygen consumption from maintenance of a mitochondrial membrane potential, the increased levels of UCP gene expression could explain the low mitochondrial membrane potential despite the high levels of mitochondrial respiration (Figure 1C) in differentiated hMSCs.

**Figure 4:**
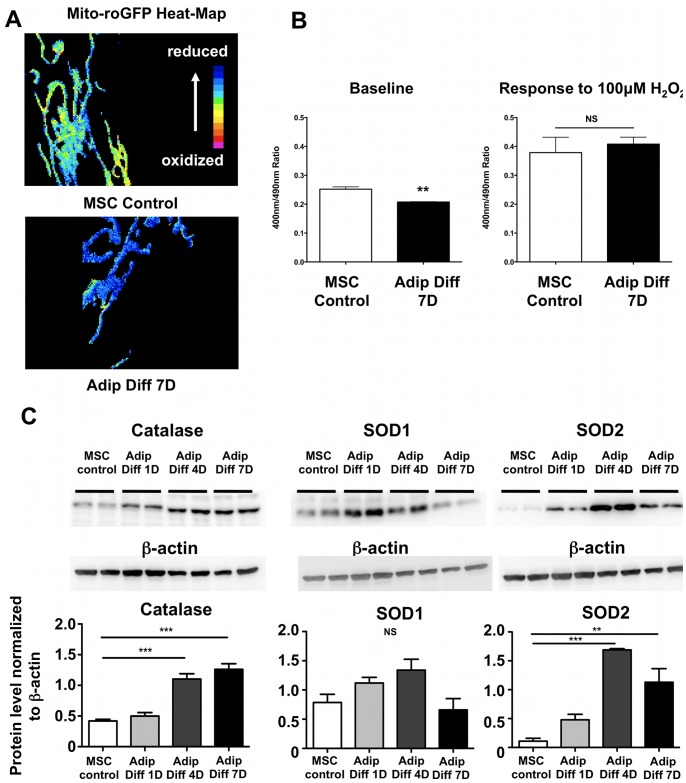
Mitochondrial redox status changes with hMSCs adipogenic differentiation. **A)** Undifferentiated and 7 day adipogenic differentiated MSCs were transfected with a redox-sensitive GFP construct (roGFP) targeted to the mitochondria. By using different excitation wavelengths (400 and 490 nm) and measuring emission at 535 nm, the redox status of cells was assessed (a higher 400/490 ratio corresponds to a more oxidized mitochondrial matrix). Ratios are represented in the form of a heat map, with reduced mitochondria shown in blue and oxidized mitochondria in red. **B)** Quantification of the roGFP data. Mitochondrial redox state is reduced after adipogenic differentiation. As a positive control, cells were also treated with 100 µM H_2_O_2_ to induce a completely oxidized state. **C)** Immunoblotting indicates that catalase and mitochondrial superoxide dismutase (SOD2) protein levels increased during differentiation, while cytoplasmic superoxide dismutase (SOD1) levels were not significantly affected by differentiation (n = 3 for each group).

Next, we checked the mitochondrial redox status directly by using a redox-sensitive GFP construct targeted to the mitochondria [19], [20], and we found that mitochondria are slightly reduced after 7 days of adipogenic differentiation compared to undifferentiated control hMSCs (Figure 4A–B). As a control, we exposed cells to 100 µM H_2_O_2_ to induce maximal oxidation of the mitochondria and we confirmed an equal signal in both groups of cells (Figure 4B). We also evaluated the protein levels of important antioxidant enzymes since upregulation of antioxidant enzymes would explain the reduced state of differentiating hMSCs. Both catalase and mitochondrial superoxide dismutase 2 (SOD2) protein levels increased significantly upon adipogenic differentiation, whereas changes in superoxide dismutase 1 (SOD1) levels were less apparent upon differentiation (Figure 4C).

### Inhibition of Mitochondrial Oxidation Prevents Adipogenic Differentiation of hMSCs

**Figure 5:**
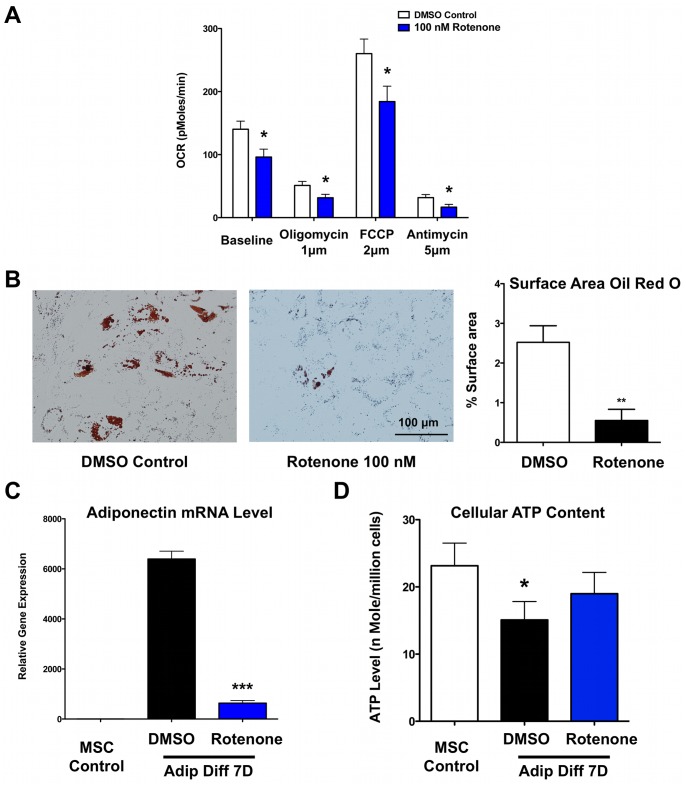
Inhibition of mitochondrial oxidation prevents adipogenic differentiation of hMSCs. **A)** 100 nM rotenone decreased the baseline oxygen consumption rate (OCR) and maximal respiration capacity (induced by FCCP treatment) in hMSCs (n = 7 for each group). **B)** Oil Red O staining showed that chronic treatment with 100 nM Rotenone inhibited adipogenic differentiation with a significant decrease in the percentage of surface area stained with Oil Red O (n = 5 for DMSO control and n = 4 for 100 nM Rotenone treatment). **C)** Real-time RT-PCR data confirm the inhibition on hMSCs adipogenic differentiation by rotenone as adiponectin levels are significantly lower after rotenone treatment (n = 3 for each group). **D)** Importantly, chronic treatment with 100 nM rotenone for 7 days during adipogenic differentiation did not result in ATP depletion, thus suggesting that the concentration of rotenone used in our studies was not toxic (n = 3 for each group).

To more specifically look at the importance of mitochondrial electron chain (ETC) activity during differentiation, we used rotenone, which specifically inhibits the transfer of electrons from iron-sulfur centers in complex I to ubiquinone [21]. In hMSCs, 24 hours treatment with 100 nM rotenone decreased oxygen consumption at baseline and after treatment with oligomycin, FCCP and antimycin compared to DMSO treated control cells (Figure 5A). After 7 days of differentiation in the presence of rotenone, a decreased uptake of Oil Red O was observed (Figure 5B). The inhibitory effect of rotenone is further confirmed by decreased adiponectin mRNA expression (Figure 5C). While higher doses of rotenone induced cell death (data not shown), we confirmed that chronic treatment with 100 nM rotenone for 7 days did not result in ATP depletion, suggesting that the rotenone levels used were non-toxic (Figure 5D).

### Hypoxia and TFAM Knockdown Inhibit Adipogenic Differentiation of hMSCs

As a more physiologically relevant stimulus to inhibit mitochondrial activity, we studied whether 1% hypoxia would similarly reduce adipogenic differentiation. Immunocytochemistry showed nuclear accumulation of HIF1α, confirming that cells are exposed to hypoxia (Figure S2A). In line with the rotenone data, we observed reduced Oil Red O staining when hMSCs were exposed to hypoxia during differentiation (Figure S2B) and adiponectin mRNA levels were similarly reduced (Figure S2C).

**Figure 6:**
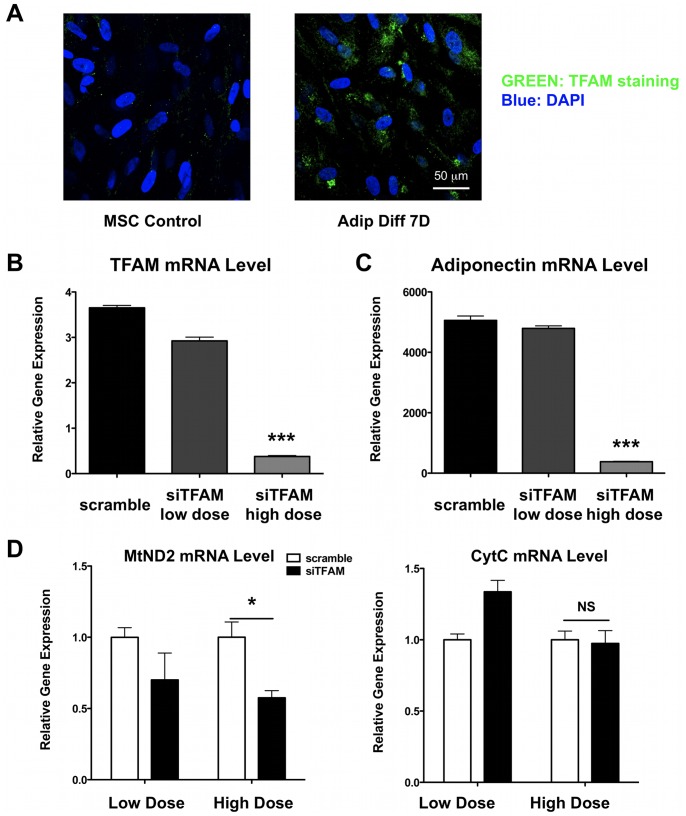
TFAM knockdown inhibits adipogenic differentiation of hMSCs. **A)** Immunofluorescent staining for TFAM, the key regulator of mitochondrial transcription, showed that TFAM is upregulated in hMSCs undergoing adipogenic differentiation. **B)** High dose siTFAM can significantly lower TFAM expression levels (n = 4 for each group). **C)** Knockdown of TFAM inhibits the differentiation process as confirmed by lower adiponectin mRNA levels (n = 4 for each group). **D)** Lowering TFAM results in lower expression of the mitochondrial gene MtND2, while the nuclear gene cytochrome C (CytC) is not affected, confirming the specificity of siRNA treatment (n = 3 for each group).

Since hypoxia influences multiple pathways in the cell, we also used a specific genetic knockdown approach. Transcription factor A, mitochondrial (TFAM) is a crucial activator of mitochondrial transcription and genome duplication. We observed increased TFAM staining after 7 days of adipogenic differentiation (Figure 6A). Interestingly, when we suppressed TFAM expression with small interfering RNA (Figure 6B), hMSC adipogenic differentiation was inhibited as adiponectin mRNA levels were significantly lower (Figure 6C). To confirm the specificity of the knockdown, we observed that the expression of the TFAM-dependent mitochondrial gene MtND2 was significantly lowered but the expression of the TFAM-independent gene CytC was not changed (Figure 6D).

## Discussion

In our present study, we demonstrate that during human MSC adipogenic differentiation, mitochondrial biogenesis and cellular oxygen consumption were increased compared to undifferentiated control cells, while lactate production was decreased (Figures 1–2). This suggests a metabolic switch during hMSC differentiation which depends on increased mitochondrial activity. Interestingly, we observed mitochondrial membrane depolarization and increased UCP1, UCP2, and UCP3 mRNA expression levels in the differentiated cells (Figure 3). Mitochondrial uncoupling leads to mitochondrial membrane depolarization and results in oxygen consumption without generating ATP. UCP1 was first described to play a role in heat generation in mammalian brown fat [22] and the increase in UCP1 mRNA levels may thus indicate that a significant fraction of the newly differentiated adipocytes exhibit a brown-fat phenotype. Using a mitochondria-targeted redox-sensitive GFP construct, we further observed that differentiated hMSCs have a reduced cellular oxidation state, which possibly can be explained by the induction of catalase and superoxide dismutase 2 (Figure 4). To more directly assess the importance of mitochondria during differentiation, we used 3 complementary approaches. First, we differentiated MSCs in the presence of rotenone, a well-known mitochondrial complex I inhibitor, and showed that reducing mitochondrial electron transport activity inhibited adipogenic differentiation (Figure 5). Second, we used 1% hypoxia as a more physiological approach to inhibit mitochondrial activity and we found that hypoxia to a similar extent inhibits adipogenic differentiation (Figure S2). Third, to confirm the importance of mitochondria with greater specificity, we used siRNA-mediated knockdown of TFAM. We confirmed that knocking down TFAM specifically lowers mitochondrial gene expression and prevents adipogenic differentiation (Figure 6). Together, these data unequivocally demonstrate that mitochondrial respiration is a prerequisite for adipogenic differentiation to occur.

Mitochondrial biogenesis has been shown to be essential for adipogenic differentiation of the 3T3 mouse embryonic fibroblast cell line and proteomic analysis confirmed a specific upregulation of mitochondrial proteins and a switch towards oxidative metabolism upon differentiation [23]. Moreover, adipogenic differentiation of hMSCs is also associated with an increase in electron transport complexes in the mitochondria [14], [24]. However, to our knowledge, this is the first demonstration that three distinct approaches to suppress mitochondrial respiration - pharmacologic inhibition, hypoxic inhibition and targeted suppression of mitochondrial transcription - yield concordant results, showing that mitochondrial biogenesis is essential for adipogenic differentiation. Interestingly, a similar increase in mitochondrial biogenesis has been observed in spontaneously differentiating human embryonic stem cells [25] and is necessary for efficient cardiac differentiation [26]. Therefore, it appears that mitochondrial biogenesis and increased mitochondrial respiration is likely a general hallmark of stem cell differentiation in adult and embryonic stem cells. Although speculative at this point, a possible explanation is that stem cells are normally maintained in a niche where they are less dependent on functional mitochondria for energy or metabolic intermediates and that mitochondrial mass needs to increase once stem cells start to differentiate.

Despite increased oxygen consumption, ATP levels decreased upon adipogenic differentiation (Figure 1E). The lower levels of ATP are probably related to increased expression of the uncoupling proteins UCP 1/2/3 (Figure 3B), which uncouple oxygen consumption from ATP production. We also cannot rule out that adipocytes consume significant amounts of ATP to generate fatty acids and hence have lower steady state levels of ATP. Interestingly, these findings contrast with increased ATP levels during osteogenic differentiation [13].

Importantly, upon long term culture, MSCs lose their differentiation capacity in part because of increased ROS formation and decreased anti-oxidant capacity [27] and therefore we only used early passage hMSCs in our studies (passage 4–5).

The methods used to block mitochondrial activity are different for each of the 3 treatments that we used. Rotenone blocks complex I of the electron transport chain, but can have possible toxic side effects. Therefore, we performed a dose-response for rotenone and selected a dose of 100 nM because it is well tolerated during long term culture and does not lead to cell death or decreased ATP levels (Figure 5D). Hypoxia blocks the conversion of pyruvate into acetyl-CoA by inhibiting PDH [28]. A recent publication has shown that hypoxia (1% oxygen) impairs osteogenic differentiation of hMSCs [29], which is in line with our findings of hypoxia reducing adipogenic differentiation of hMSCs (Figure S2). Hypoxia leads to the regulation of many genes and therefore is not a specific inhibitor of mitochondrial function. In contrast, TFAM is a transcription factor that binds to the mitochondrial genome and can induce replication of the mitochondrial genome and is essential for increasing mitochondrial gene expression. TFAM protein expression is increased with hMSC adipogenic differentiation (Figure 6A), suggesting that the observed increase in mitochondrial mass during differentiation was indeed a result of increased biogenesis and not just due to reduced mitochondrial degradation. Combined, our 3 approaches to inhibit mitochondrial function make a strong case that increased mitochondrial function is necessary for successful adipogenic differentiation of hMSCs.

Mitochondria generate ATP, contain enzymes for the Krebs cycle and are an important source of ROS and it is currently not clear whether a specific mitochondrial function is essential for adipogenic differentiation. Nevertheless, our current experiments show that upon differentiation, mitochondrial redox status becomes more reduced (Figure 4A) while catalase and superoxide dismutase 2 levels are increased (Figure 4C), suggesting that an increase in ROS is not necessary for differentiation to occur. In fact, increased ROS levels in MSCs have been associated with reduced adipogenic differentiation potential [27].

In our study, we decided to focus on adipogenic differentiation of hMSCs, but other studies have found similar results in osteogenic differentiation. For example, during osteogenic differentiation a similar shift from glycolysis to aerobic metabolism occurs which is in line with our findings [13]. Moreover, increased mitochondrial biogenesis and catalase/SOD2 upregulation were also observed during osteogenic differentiation [13]. A high mitochondrial membrane potential is associated with better osteogenic differentiation potential of hMSCs, suggesting that having functional mitochondria is a prerequisite for efficient osteogenic differentiation [30]. However, in our manuscript, we show for the first time that metabolic changes and upregulation of mitochondrial proteins occurs during adipogenic differentiation. Moreover, we have directly addressed the importance of mitochondria in the differentiation process by using 3 complementary and specific approaches to inhibit mitochondrial function.

An important characteristic of MSC based therapies is that some of their therapeutic benefits depend on the release of beneficial paracrine factors by undifferentiated MSCs [31]. Maintaining the undifferentiated MSC state following transplantation and avoiding the differentiation into undesired lineages such as the formation of adipose cells in the heart may be necessary. Our findings would suggest that targeted inhibition of mitochondria could help maintain the undifferentiated state for such paracrine therapies.

The inhibitory effect of hypoxia on adipogenic differentiation may also help explain the high-altitude weight loss that has been observed in obese humans [32]. Although subcutaneous or abdominal adipocytes in obesity are formed from pre-adipocytes and not from bone marrow MSCs, it is likely that the mechanisms we observed in adipogenic differentiation of MSCs are also critical for adipogenesis in obesity. Being able to manipulate the formation of adipocytes by specifically suppressing mitochondrial respiration may be an important therapeutic approach that could be explored as a treatment for obesity.

## Limitations

Our findings demonstrate that mitochondrial metabolism is required for adipogenic differentiation, but additional work is needed to identify specific molecular mechanisms by which mitochondrial respiration affects adipogenesis. A previous study suggested that the release of mitochondrial ROS may be required for adipogenic differentiation of MSCs [24], however we did not observe any significant increase in ROS during differentiation. If anything, our results showed a marked increase in the anti-oxidant defense enzymes catalase and SOD2 and an overall reduction in the mitochondrial redox status. One potential explanation to reconcile our findings and the previous work [24] is that mitochondrial ROS may be released early on in the differentiation process and that when we assessed the redox state (day 7 of differentiation), differentiating cells had already responded to the mitochondrial ROS by increasing antioxidant enzyme expression.

## Conclusions

Collectively, our present study indicates that mitochondrial biogenesis and metabolism are necessary for the adipogenic differentiation of human MSCs. This points to the important active role that mitochondrial metabolism plays in the differentiation of adult stem cells [33] and that metabolic cues or metabolic niches are perhaps as critical for successful stem cell differentiation as commonly used differentiation cues, such as growth factors.

## Supporting Information

Figure S1 **Pyruvate dehydrogenase (PDH) is increased upon adipogenic differentiation.** PDH immunocytochemistry showed that differentiated cells have a higher content of PDH compared to undifferentiated hMSCs.
(TIF)

Figure S2 **Hypoxia exposure can inhibit adipogenic differentiation of hMSCs.** **A)** Hypoxia is a potent suppressor of mitochondrial oxidation. To evaluate the effect of hypoxia on hMSC differentiation, we first confirmed that 1% O_2_ was adequate to activate hypoxia inducible factor 1-alpha (HIF1α) in hMSCs. **B–C)** We then studied the effect of chronic hypoxia on adipogenic differentiation in hMSCs. As determined by both Oil Red O staining (Panel B) and gene expression analysis (Panel C), hypoxia was a potent suppressor of adipogenic differentiation.
(TIF)

Table S1 Primer sequences used for real-time RT-PCR.
(DOC)

## References

[1] Friedenstein AJ, Gorskaja JF, Kulagina NN (1976) Fibroblast precursors in normal and irradiated mouse hematopoietic organs. Exp Hematol 4: 267–274. 976387

[2] Friedenstein AJ, Deriglasova UF, Kulagina NN, Panasuk AF, Rudakowa SF, et al (1974) Precursors for fibroblasts in different populations of hematopoietic cells as detected by the in vitro colony assay method. Exp Hematol 2: 83–92. 4455512

[3] Islam MN, Das SR, Emin MT, Wei M, Sun L, et al (2012) Mitochondrial transfer from bone-marrow-derived stromal cells to pulmonary alveoli protects against acute lung injury. Nature medicine 18: 759–765. 10.1038/nm.2736 PMC3727429 22504485

[4] Toma C, Pittenger MF, Cahill KS, Byrne BJ, Kessler PD (2002) Human mesenchymal stem cells differentiate to a cardiomyocyte phenotype in the adult murine heart. Circulation 105: 93–98. 11772882 10.1161/hc0102.101442

[5] Amado LC, Saliaris AP, Schuleri KH, St John M, Xie JS, et al (2005) Cardiac repair with intramyocardial injection of allogeneic mesenchymal stem cells after myocardial infarction. Proc Natl Acad Sci U S A 102: 11474–11479. 16061805 10.1073/pnas.0504388102 PMC1183573

[6] Valina C, Pinkernell K, Song YH, Bai X, Sadat S, et al (2007) Intracoronary administration of autologous adipose tissue-derived stem cells improves left ventricular function, perfusion, and remodelling after acute myocardial infarction. Eur Heart J 28: 2667–2677. 17933755 10.1093/eurheartj/ehm426

[7] Silva GV, Litovsky S, Assad JA, Sousa AL, Martin BJ, et al (2005) Mesenchymal stem cells differentiate into an endothelial phenotype, enhance vascular density, and improve heart function in a canine chronic ischemia model. Circulation 111: 150–156. 15642764 10.1161/01.CIR.0000151812.86142.45

[8] Zhang DX, Gutterman DD (2007) Mitochondrial reactive oxygen species-mediated signaling in endothelial cells. Am J Physiol Heart Circ Physiol 292: H2023–2031. 17237240 10.1152/ajpheart.01283.2006

[9] McBride HM, Neuspiel M, Wasiak S (2006) Mitochondria: more than just a powerhouse. Curr Biol 16: R551–560. 16860735 10.1016/j.cub.2006.06.054

[10] Carriere A, Ebrahimian TG, Dehez S, Auge N, Joffre C, et al (2009) Preconditioning by mitochondrial reactive oxygen species improves the proangiogenic potential of adipose-derived cells-based therapy. Arterioscler Thromb Vasc Biol 29: 1093–1099. 19423864 10.1161/ATVBAHA.109.188318

[11] Varum S, Rodrigues AS, Moura MB, Momcilovic O, Easley CAt, et al (2011) Energy metabolism in human pluripotent stem cells and their differentiated counterparts. PLoS One 6: e20914. 21698063 10.1371/journal.pone.0020914 PMC3117868

[12] Mandal S, Lindgren AG, Srivastava AS, Clark AT, Banerjee U (2011) Mitochondrial function controls proliferation and early differentiation potential of embryonic stem cells. Stem Cells 29: 486–495. 21425411 10.1002/stem.590 PMC4374603

[13] Chen CT, Shih YR, Kuo TK, Lee OK, Wei YH (2008) Coordinated changes of mitochondrial biogenesis and antioxidant enzymes during osteogenic differentiation of human mesenchymal stem cells. Stem Cells 26: 960–968. 18218821 10.1634/stemcells.2007-0509

[14] Hofmann AD, Beyer M, Krause-Buchholz U, Wobus M, Bornhauser M, et al (2012) OXPHOS supercomplexes as a hallmark of the mitochondrial phenotype of adipogenic differentiated human MSCs. PLoS One 7: e35160. 22523573 10.1371/journal.pone.0035160 PMC3327658

[15] Acquistapace A, Bru T, Lesault PF, Figeac F, Coudert AE, et al (2011) Human mesenchymal stem cells reprogram adult cardiomyocytes toward a progenitor-like state through partial cell fusion and mitochondria transfer. Stem Cells 29: 812–824. 21433223 10.1002/stem.632 PMC3346716

[16] Sekiya I, Larson BL, Smith JR, Pochampally R, Cui JG, et al (2002) Expansion of human adult stem cells from bone marrow stroma: conditions that maximize the yields of early progenitors and evaluate their quality. Stem Cells 20: 530–541. 12456961 10.1634/stemcells.20-6-530

[17] Ferrick DA, Neilson A, Beeson C (2008) Advances in measuring cellular bioenergetics using extracellular flux. Drug Discov Today 13: 268–274. 18342804 10.1016/j.drudis.2007.12.008

[18] Marsboom G, Wietholt C, Haney CR, Toth PT, Ryan JJ, et al (2012) Lung (1)(8)F-fluorodeoxyglucose positron emission tomography for diagnosis and monitoring of pulmonary arterial hypertension. Am J Respir Crit Care Med 185: 670–679. 22246173 10.1164/rccm.201108-1562OC PMC3326289

[19] Dooley CT, Dore TM, Hanson GT, Jackson WC, Remington SJ, et al (2004) Imaging dynamic redox changes in mammalian cells with green fluorescent protein indicators. J Biol Chem 279: 22284–22293. 14985369 10.1074/jbc.M312847200

[20] Rehman J, Zhang HJ, Toth PT, Zhang Y, Marsboom G, et al (2012) Inhibition of mitochondrial fission prevents cell cycle progression in lung cancer. FASEB J 26: 2175–2186. 22321727 10.1096/fj.11-196543 PMC3336787

[21] Barrientos A, Moraes CT (1999) Titrating the effects of mitochondrial complex I impairment in the cell physiology. J Biol Chem 274: 16188–16197. 10347173 10.1074/jbc.274.23.16188

[22] Bouillaud F, Ricquier D, Mory G, Thibault J (1984) Increased level of mRNA for the uncoupling protein in brown adipose tissue of rats during thermogenesis induced by cold exposure or norepinephrine infusion. J Biol Chem 259: 11583–11586. 6470011

[23] Wilson-Fritch L, Burkart A, Bell G, Mendelson K, Leszyk J, et al (2003) Mitochondrial biogenesis and remodeling during adipogenesis and in response to the insulin sensitizer rosiglitazone. Mol Cell Biol 23: 1085–1094. 12529412 10.1128/MCB.23.3.1085-1094.2003 PMC140688

[24] Tormos KV, Anso E, Hamanaka RB, Eisenbart J, Joseph J, et al (2011) Mitochondrial complex III ROS regulate adipocyte differentiation. Cell Metab 14: 537–544. 21982713 10.1016/j.cmet.2011.08.007 PMC3190168

[25] Cho YM, Kwon S, Pak YK, Seol HW, Choi YM, et al (2006) Dynamic changes in mitochondrial biogenesis and antioxidant enzymes during the spontaneous differentiation of human embryonic stem cells. Biochem Biophys Res Commun 348: 1472–1478. 16920071 10.1016/j.bbrc.2006.08.020

[26] Chung S, Dzeja PP, Faustino RS, Perez-Terzic C, Behfar A, et al (2007) Mitochondrial oxidative metabolism is required for the cardiac differentiation of stem cells. Nat Clin Pract Cardiovasc Med 4 Suppl 1S60–67. 17230217 10.1038/ncpcardio0766 PMC3232050

[27] Geissler S, Textor M, Kuhnisch J, Konnig D, Klein O, et al (2012) Functional comparison of chronological and in vitro aging: differential role of the cytoskeleton and mitochondria in mesenchymal stromal cells. PLoS One 7: e52700. 23285157 10.1371/journal.pone.0052700 PMC3532360

[28] Kim JW, Tchernyshyov I, Semenza GL, Dang CV (2006) HIF-1-mediated expression of pyruvate dehydrogenase kinase: a metabolic switch required for cellular adaptation to hypoxia. Cell Metab 3: 177–185. 16517405 10.1016/j.cmet.2006.02.002

[29] Hsu SH, Chen CT, Wei YH (2013) Inhibitory Effects of Hypoxia on Metabolic Switch and Osteogenic Differentiation of Human Mesenchymal Stem Cells. Stem cells Epub Jun 4 2013. 10.1002/stem.1441 23733376

[30] Pietila M, Lehtonen S, Narhi M, Hassinen IE, Leskela HV, et al (2010) Mitochondrial function determines the viability and osteogenic potency of human mesenchymal stem cells. Tissue Eng Part C Methods 16: 435–445. 19839730 10.1089/ten.tec.2009.0247

[31] Psaltis PJ, Zannettino AC, Worthley SG, Gronthos S (2008) Concise review: mesenchymal stromal cells: potential for cardiovascular repair. Stem Cells 26: 2201–2210. 18599808 10.1634/stemcells.2008-0428

[32] Lippl FJ, Neubauer S, Schipfer S, Lichter N, Tufman A, et al (2010) Hypobaric hypoxia causes body weight reduction in obese subjects. Obesity (Silver Spring) 18: 675–681. 20134417 10.1038/oby.2009.509

[33] Rehman J (2010) Empowering self-renewal and differentiation: the role of mitochondria in stem cells. J Mol Med (Berl) 88: 981–986. 20809088 10.1007/s00109-010-0678-2 PMC3006229

