# Supplementary figures and images for: Mitochondrial Respiration Regulates Adipogenic Differentiation of Human Mesenchymal Stem Cells
Source: PLoS One. 2013 Oct 18;8(10):e77077. doi: 10.1371/journal.pone.0077077 (PMC3800007; doi:10.1371/journal.pone.0077077)

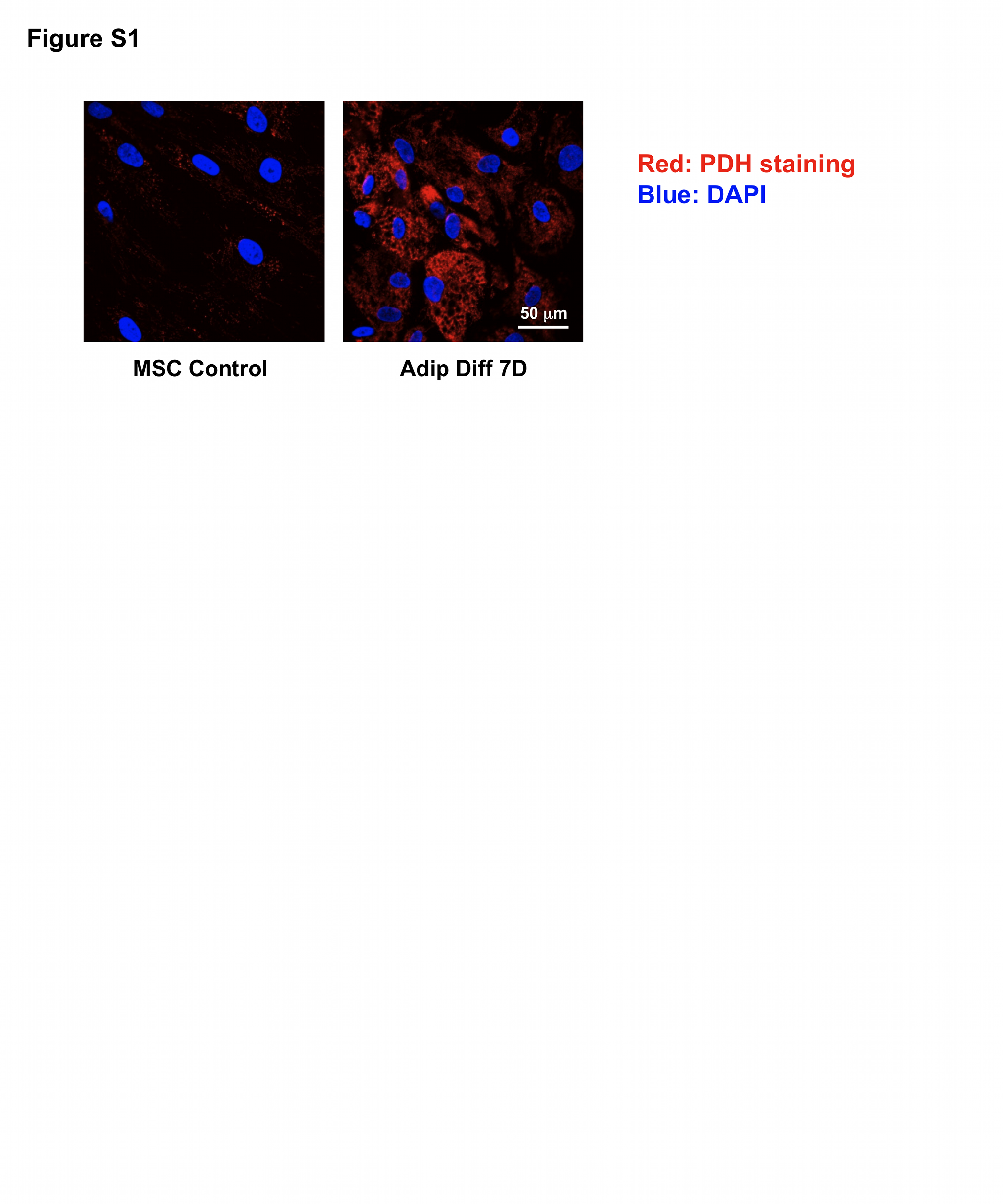

Supplement: Figure S1 — Pyruvate dehydrogenase (PDH) is increased upon adipogenic differentiation. PDH immunocytochemistry showed that differentiated cells have a higher content of PDH compared to undifferentiated hMSCs. (TIF) [file pone.0077077.s001.tif]

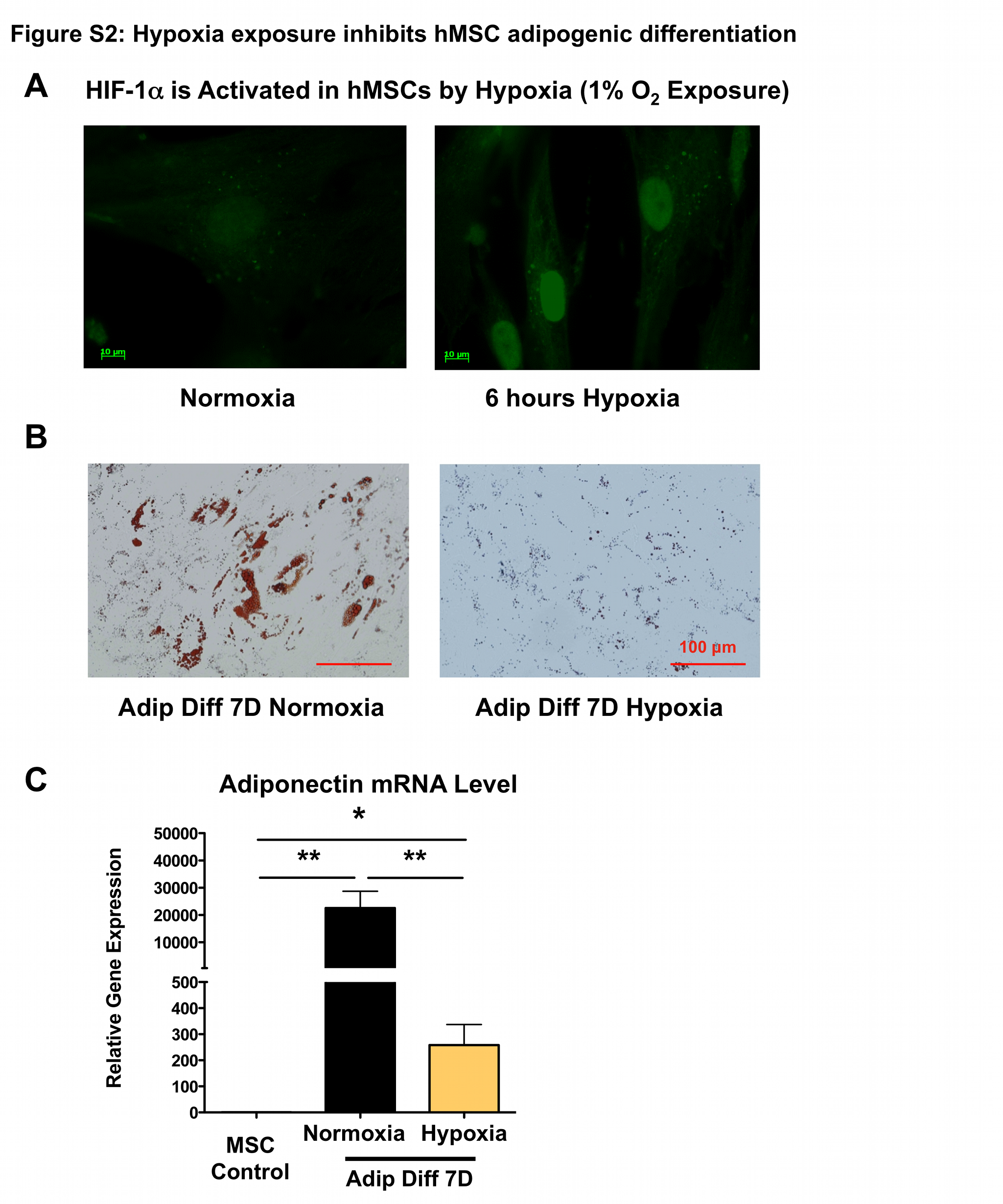

Supplement: Figure S2 — Hypoxia exposure can inhibit adipogenic differentiation of hMSCs. A) Hypoxia is a potent suppressor of mitochondrial oxidation. To evaluate the effect of hypoxia on hMSC differentiation, we first confirmed that 1% O2 was adequate to activate hypoxia inducible factor 1-alpha (HIF1α) in hMSCs. B–C) We then studied the effect of chronic hypoxia on adipogenic differentiation in hMSCs. As determined by both Oil Red O staining (Panel B) and gene expression analysis (Panel C), hypoxia was a potent suppressor of adipogenic differentiation. (TIF) [file pone.0077077.s002.tif]
