# Supplementary material for: Mitochondrial Respiration Regulates Adipogenic Differentiation of Human Mesenchymal Stem Cells
Source: PLoS One. 2013 Oct 18;8(10):e77077. doi: 10.1371/journal.pone.0077077 (PMC3800007; doi:10.1371/journal.pone.0077077)
Supplement: Table S1 — Primer sequences used for real-time RT-PCR. (DOC) [file pone.0077077.s003.doc]

**Table S1: RT-PCR primer sequences**

| **Gene** | **Orientation** | **Sequence** |
| --- | --- | --- |
| Adiponectin | Forward | TGCCCAAAGAGGAGAGAGGAA |
|  | Reverse | TCAGAAACAGGCACACAACTCA |
| Cytochrome C | Forward | GATACTCTTACACAGCCGCCAA |
|  | Reverse | TCTGCCCTTTCTTCCTTCTTCTT |
| PGC1α | Forward | ACAGTCGCAGTCACAACACTTACA |
|  | Reverse | CACACTTAAGGTGCGTTCAATAGTC |
| TFAM | Forward | GAACCCAGATGCAAAAACTACAGA |
|  | Reverse | CACTCCGCCCTATAAGCATCTT |
| UCP1 | Forward | CAAATCAGCTCCGCCTCTCT |
|  | Reverse | AATGAATACTGCCACTCCTCCAG |
| UCP2 | Forward | CCCTCCTGAAAGCCAACCTC |
|  | Reverse | AGAAGCCTGCCCCAAAGG |
| UCP3 | Forward | AACCATCGCCAGGGAGGA |
|  | Reverse | GGGAAGTTGTCAGTGAGCAGGT |
